# Case report: Primary alveolar soft-part sarcoma of the lung in a child

**DOI:** 10.3389/fsurg.2023.927597

**Published:** 2023-02-20

**Authors:** Zhufei Xu, Jinhu Wang, Junqing Mao, Dan Xu, Lei Wu, Yunlian Zhou, Xuejing Li, Zhimin Chen, Yingshuo Wang

**Affiliations:** ^1^ Department of Pulmonology, Children's Hospital, Zhejiang University School of Medicine, National Clinical Research Center for Child Health, Hangzhou, China; ^2^ Department of Oncology, Children's Hospital, Zhejiang University School of Medicine, National Clinical Research Center for Child Health, Hangzhou, China

**Keywords:** alveolar soft-part sarcoma, lung, antiangiogenic agent, child, case report, TFE3

## Abstract

Alveolar soft-part sarcoma involving the lung is mostly metastatic in nature, while primary alveolar soft-part sarcoma involving the lung occurs more rarely. Herein, we report a rare case of a patient with primary alveolar soft-part sarcoma of the lung, which may represent the earliest onset of this condition reported thus far. In this patient, surgery was performed to excise the lesion to the greatest extent possible, and the combination of surgery with chemoradiotherapy and an antiangiogenic agent may provide an important reference for the development of standard or first-line treatment for such pediatric patients.

## Introduction

Alveolar soft-part sarcoma (ASPS) is a rare sarcoma of soft tissue characterized by ASPSCR1–TFE3 gene fusion (1, 2). When ASPS is diagnosed, it is usually metastatic in nature, and it is prone to lung, bone, and brain metastases, leading to a poor long-term prognosis. ASPS involving the lung is mostly metastatic, while primary ASPS involving the lung occurs rarely (3–5). At present, there are only a few cases of patients affected by this condition, and therefore, no standard or first-line recommended treatment has been proposed. Herein, we report a rare case of a child with primary ASPS of the lung; this case may represent the earliest onset of such ASPS reported thus far and the only case treated via a new molecular targeted therapy with an antiangiogenic agent.

## Case presentation

A 7-year-old female child was admitted to our hospital on April 20, 2020, due to foreign body aspiration of corn that occurred 2 days prior to the development of a cough and shortness of breath. A local hospital chest computed tomography (CT) revealed a strong possibility of the presence of a foreign body in the bronchus; the patient was therefore hospitalized.

At the age of 3 years, the child had been diagnosed with thalassemia (mild), with no history of any prior blood transfusions nor of recurrent cough and wheeze. She also had no family history of malignancy.

Physical examination on admission revealed: temperature 37°C, heart rate 100 beats/min, respiration 26 breaths/min, blood pressure 101/53 mmHg, weight 20.8 kg, clear consciousness, steady breathing, coarse breath sounds on auscultation, no obvious dry or wet rales. Auxiliary examinations of chest CT conducted in our hospital revealed: (1) stenosis of the right middle and lower lobe bronchus with a high-density shadow, suggesting the presence of a foreign body, with right middle and lower lobe obstructive emphysema; (2) the upper lobe of the right lung scattered with fuzzy patchy shadow, suggesting inflammation. Primary diagnosis suggested the presence of a pulmonary foreign body and bronchopneumonia.

**Figure 1:**
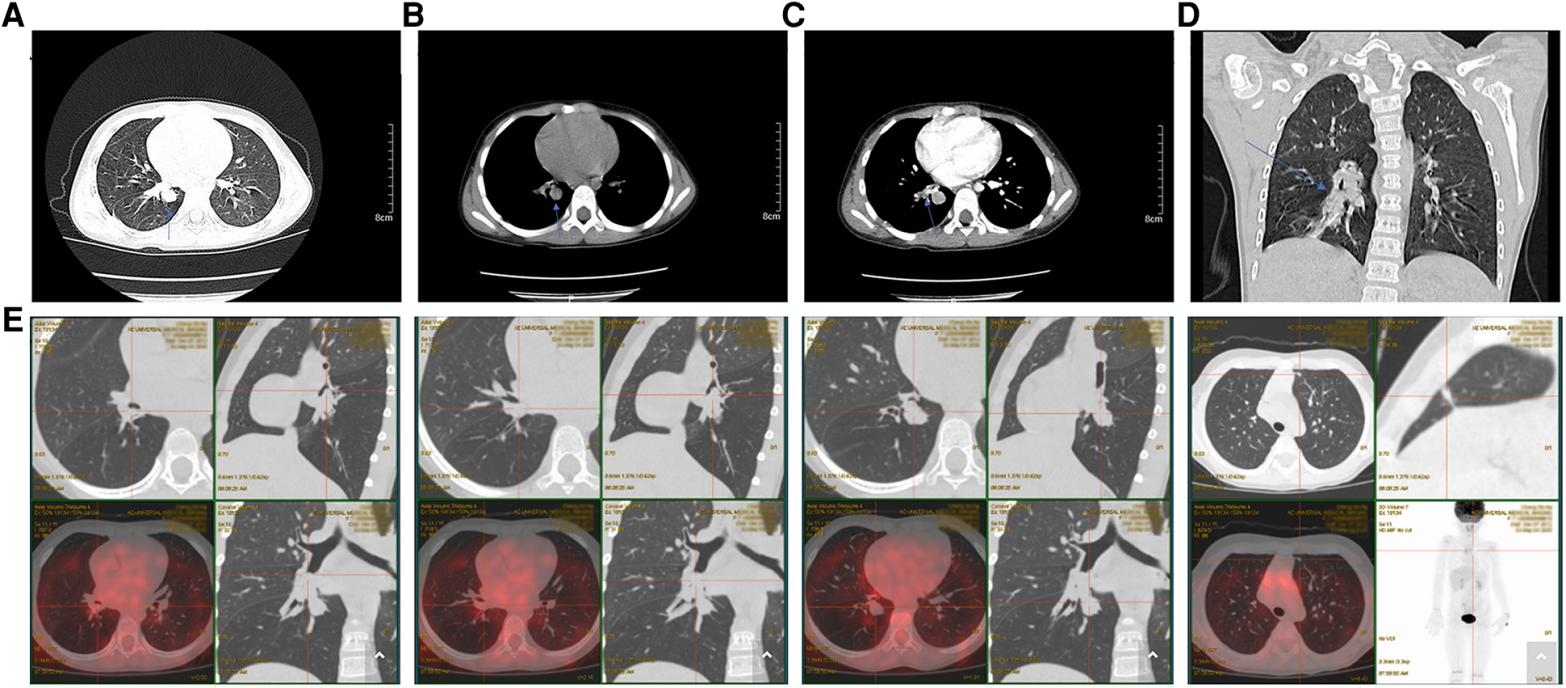
(**A**) A round mass in the right lower lobe adjacent to the bronchus, with a well-defined boundary and thickened adjacent lung texture (blue arrow). (**B**) A mediastinal window on the same plane as the image above shows a round soft tissue mass adjacent to the bronchus in the lower lobe of the right lung, with a well-defined boundary and thickened adjacent lung texture (blue arrow). (**C**) Contrast-enhanced mediastinal window scan on the same plane shows obvious enhancement of a round soft tissue mass adjacent to the bronchus in the lower lobe of the right lung, with uniform enhancement, a well-defined boundary, no burr or satellite foci, and an irregular soft tissue mass near the lung texture (blue arrow). (**D**) Coronal reconstruction, showing irregular density shadow in the lower lobe of the right lung with a well-defined boundary, surrounding the bronchial branches of the lower lobe. (**E**) PET-CT: soft tissue mass in the right middle bronchus, the right lower lobe bronchus, the basal trunk, and the medial basal segmental bronchus, along with elevated FDG metabolism and combined with pathology results, this led soft tissue sarcoma to be considered. FDG: fluorodeoxyglucose; PET-CT: Positron emission tomography/computed tomography.

On day 2 after admission, a right middle bronchial mass was detected using a fiber-optic bronchoscope under sedation and local anesthesia, and a biopsy was performed; a round neoplasm approximately 1 cm × 0.6 cm in size was found, and the nature of the lesion was to be investigated. A pathological examination, combined with a morphological examination and immunohistochemistry, first revealed ASPS. Fluorescence *in situ* hybridization (FISH) also revealed ASPS first and then a TFE3-positive tumor, following which a recommendation for next-generation sequencing was made. On postoperative day 2, the chest was examined by enhanced CT (Figures 1A–D): (1) the middle bronchus of the right lung changed postoperatively, with the wall being slightly rough, and the upper portion of the lower bronchus was visible; (2) multiple paratracheal nodules in the lower lobe of the right lung were found, so enlarged lymph nodes were considered; (3) emphysema and inflammation in the middle and lower lobes of the right lung and local thickening of the right lung pleura were observed. Positron emission tomography/computed tomography (PET-CT) (Figure 1E) revealed a soft tissue mass in the lumen of the right middle bronchus, right lower lobe bronchus, basal trunk, and medial basal segmental bronchus, and increased fluorodeoxyglucose (FDG) metabolism; given these findings combined with pathology results, soft tissue sarcoma was considered.

**Figure 2:**
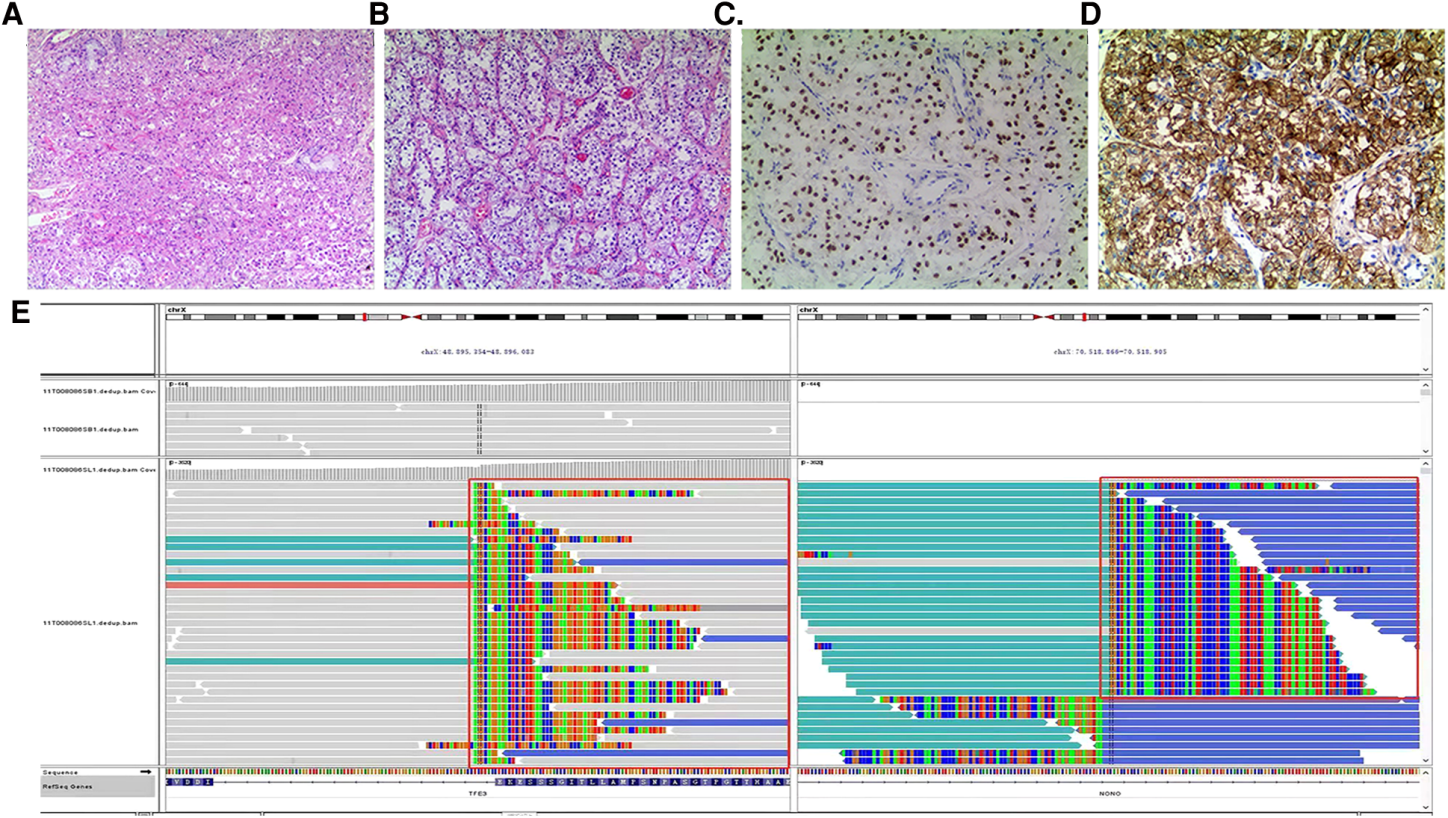
(**A,B**) (HE staining ×100) Tumor cells are arranged in a nest-like and alveolar manner, with abundant cytoplasm, some of which are eosinophilic and transparent. The nuclei are round in shape with obvious nucleoli and abundant blood supply in the tumor. (**C**) (Immunohistochemical TFE3 ×200) Tumor cells TFE3 positive. (**D**) (Immunohistochemical CD56 ×200) The cytoplasm and membrane of tumor cells positive. (**E**) (Next-generation sequencing) TFE3 rearrangement, ASPSCR1–TFE3 fusion.

**Figure 3:**
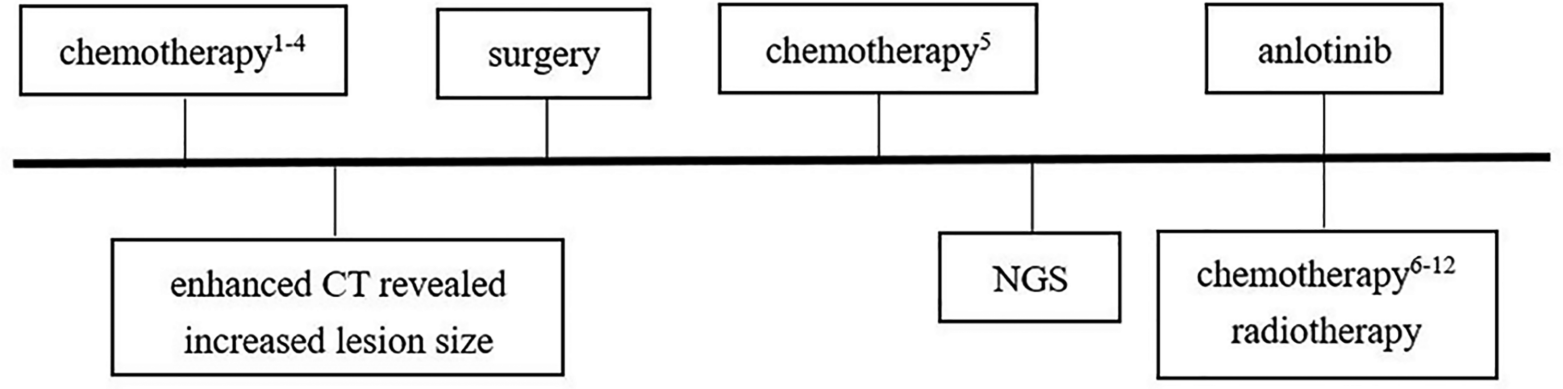
Treatment flow chart. Chemotherapy^1–4^: vindesine 3 mg/m^2^, d1 + cyclophosphamide 1.2 g/m^2^, d1 + epirubicin37.5 mg/m^2^, d1–d2 (first cycle, May 11–May 12); ifosfamide 1.8 g/m^2^, d1–d5 + etoposide 100 mg/m^2^, d1–d5 (second cycle, June 3–June 7); vindesine 3 mg/m^2^, d1 + cyclophosphamide 1.2 g/m^2^, d1 + epirubicin 37.5 mg/m^2^, d1–d2 (third cycle, June 27–June 28); paclitaxel 260 mg/m^2^, d1 + cisplatin 20 mg/m^2^, d2–d5 + ifosfamide 1.2 g/m^2^, d2–d5 (fourth cycle, July 21–July 25). Chemotherapy^5^: ifosfamide 1.5 g/m^2^, d1–d5 + irinotecan 90 mg/m^2^, d1–d3 + carboplatin 450 mg/m^2^, d2 (fifth cycle: August 21–August 25). Chemotherapy^6–12^: irinotecan 90 mg/m^2^, d1–d3 + carboplatin 450 mg/m^2^, d2 (6th–12th cycles; September 18–September 20, October 15–October 17, November 6–November 8, November 27–November 29, December 25–December 27 of 2020; January 21–January 23, February 17–February 19 of 2021).

After diagnosis of ASPS, chest enhanced CT (June 30, 2020) after three cycles of chemotherapy [vindesine + cyclophosphamide + epirubicin (May 11–May 12), ifosfamide + etoposide (June 3–June 7), vindesine + cyclophosphamide + epirubicin (June 27–June 28)] revealed a mass shadow in the middle bronchial segment of the right lung, which suggested the possibility of residual tumor. Chest enhanced CT (August 4, 2020) after the fourth cycle of chemotherapy [paclitaxel + cisplatin + ifosfamide (July 21–July 25)] revealed that the size of the lesion had increased, reflecting a weak effect of chemotherapy. On August 12, 2020, a right lower lobe excision + right intrabronchial tumor excision + bronchial anastomosis + hilar lymph node dissection were performed under general anesthesia. During the operation, we found that the tumor was located at the intersection of the middle lobe of the right lung and the lower lobe of the right lung. A tumor plug had formed in the right bronchus and extended proximally above the level of the middle bronchus of the right lung, but the plug had not invaded the bronchial wall in this area. Therefore, we removed not only the lower lobe of the right lung but also the tumor thrombus in the right bronchus beyond the lower lobe of the right lung; we then performed anastomosis between the right and middle bronchi of the right lung. During the operation, we also found several obvious enlarged lymph nodes in the hilus of the right lung. At that time, we could not confirm the nature of these lymph nodes, so the visible lymph nodes were removed for pathological examination. Postoperative pathology (Figures 2A,B) revealed a grayish-white mass with a size of 1.8 cm × 1.5 cm × 1 cm in the section of the lower lobe of the right lung. The tumor cells were arranged in the shape of nests and alveoli, with abundant cytoplasm, some of which were eosinophilic and transparent. The nuclei were round with obvious nucleoli and abundant blood supply in the tumor. The resection margins of the lung tissue and bronchus were negative. In the two parabronchial lymph nodes and four intrapulmonary lymph nodes, no tumor metastasis was found. Tumor metastasis was not observed in any of the tracheal walls, and the surgical margin was considered negative resection margin (R0). Immunohistochemistry showed the following: TFE3+ (Figure 2C), CD56 + (Figure 2D), SMA −, CK (Pan) −, EMA −, SALL4 −, MyOD1 −, presence of INI1, desmin −, synaptophysin −, S-100 partial +, Ki-67 close to 10% +, MelanA −, CgA −, PHOX2B −. Combining these findings with morphology and immunohistochemistry, ASPS was first considered; no tumor metastasis was observed. FISH suggested TFE3 gene breaking (+), and therefore, a fifth cycle of the chemotherapy regimen (ifosfamide + irinotecan + carboplatin, August 21–August 25) was performed. Next-generation sequencing (Figure 2E, on August 26, 2020) revealed TFE3 rearrangement and ASPSCR1–TFE3 gene fusion. Subsequently, the 6th to 12th cycles of chemotherapy (irinotecan + carboplatin: September 18–September 20, October 15–October 17, November 6–November 8, November 27–November 29, and December 25–December 27 of 2020; January 21–January 23 and February 17–February 19 of 2021) were performed, for a total of 12 cycles of chemotherapy throughout the course of treatment (Figure 3) and a total of 28 cycles of radiotherapy (October 6–November 11, 2020). With this, chemoradiotherapy was completed. On September 18, an antiangiogenic agent, anlotinib, was started; this treatment has continued up to the time of writing. Oral treatment with anlotinib was given at a dosage of 8 mg once per day, taken for 14 days and then stopped for 7 days. The course of anlotinib is expected to continue for approximately 2 years, during which time side effects and tumor enlargement and metastasis will be monitored. The final diagnosis was malignant neoplasm (primary alveolar soft tissue sarcoma of the right lung, TFE3 gene breaking, stage III). At present, after more than 8 months of postoperative follow-up, no tumor enlargement or metastasis has been observed [under multiple reexaminations of enhanced CT, cranial magnetic resonance imaging (MRI), and ultrasound of the abdomen and retroperitoneum]. At the time of writing, no obvious drug-related side effects have occurred in the patient.

## Discussion

Primary pulmonary ASPS is a rare condition, with only three cases being reported thus far (3–5). Herein, we report on another case that represents possibly the earliest onset of this condition reported to date (Table 1). A diagnosis of primary pulmonary ASPS should exclude secondary ASPS. Therefore, our patient underwent PET-CT, which did not reveal tumor foci in other locations. In a prospective trial, Brennan et al. (6) reported on the case of a 2.7-year-old patient with ASPS, who was younger than our patient. However, in all patients who were enrolled into the trial the sites of origin of primary tumor were the extremities, trunk, head, and neck. TFE3 testing was positive in two cases, while such testing was not described in the other two cases. PAS staining was positive in two cases and was also not described in the other two. One patient did not undergo surgical treatment, while three underwent surgical excision, two of whom were treated with chemotherapy and two with radiotherapy. The antiangiogenic drug anlotinib was used only in the case of our patient. The only fatality was a 49-year-old man treated with only two cycles of palliative chemotherapy without surgery due to rapidly progressive disease and significant respiratory distress. In this case, death occurred only 1 month after hospitalization.

**Table 1:** Statistics on four cases of patients with primary pulmonary ASPS.

Author	Gender	Age (years)	Comorbidity	Symptoms	Tumor size (cm)	Metastasis/site if yes	Surgery	Chemotherapy	Radiotherapy	New targeted drug	PAS	TFE3	Death	Follow-up period (months)
Zhao (3)	Female	48	No	No	3.5 × 3.2 × 3.0	No	Yes	—	—	—	—	(+)	No	12
Trabelsi (4)	Male	49	No	Thoracic pain	6.0 × 5.0 × 5.0	No	No	Yes	—	—	(+)	/	Yes	1
Kim (5)	Female	42	Thyroid nodule	No	3.8 × 3.5 × 3.5	Yes/lumbar spine and occipital scalp	Yes	—	Yes	—	(+)	/	No	25
Our case	Female	7	Thalassemia	Cough and shortness of breath	1.8 × 1.5 × 1.0	No	Yes	Yes	Yes	Yes	—	(+)	No	8+

ASPS: alveolar soft-part sarcoma.

Currently, the recommended treatment is radical surgical excision of the primary tumor and metastatic lesions (7), which is the preferred treatment for ASPS. The combination of radiotherapy and surgery has not been accepted due to a lack of evidence that this could improve the survival rate (8). Nevertheless, if the tumor cannot be completely excised, systemic treatment is still recommended. Clinical studies have shown that molecular targeted agents, especially antiangiogenic agents, are more effective for ASPS. Antiangiogenic therapies provide great clinical utility for the ASPS-rich vascular system. The newly identified ASPS is characterized by a specific oncogenic translocation of ASPSCR1-TFE3, which induces overexpression of hepatocyte growth factor receptor (MET), angiogenesis, and immunosuppression in the tumor microenvironment. These specific biological characteristics encourage the exploration of MET inhibitors, antiangiogenic agents, and immunotherapy in the treatment of ASPS (9, 10). Specifically, this may consist of: (1) MET inhibitors: tivantinib, crizotinib; (2) antiangiogenic therapies: bevacizumab, sunitinib, pazopanib, cediranib, cabozantinib, anlotinib; (3) immunotherapy: atezolizumab, pembrolizumab, and so on. Paoluzzi and Maki (11) reviewed the literature regarding ASPS and tyrosine kinase inhibitors and found that the use of tyrosine kinase inhibitors, such as sunitinib, cediranib, and pazopanib, led to notable clinical outcomes in patients with ASPS.

Anlotinib is a highly selective multitarget tyrosine kinase inhibitor that has been independently developed in China and can potently inhibit multiple targets, achieving the effects of antitumor angiogenesis and inhibition of tumor cell growth (12). In June 2019, anlotinib was officially approved for the treatment of ASPS in China, thus becoming the first approved drug for this condition (13). The 2019 CSCO guidelines for the management and treatment of soft tissue sarcoma recommend anlotinib as a second-line treatment strategy for advanced or unexcisable soft tissue sarcoma. Possible side effects of anlotinib primarily include hypertension, elevated triglycerides, elevated thyroid-stimulating hormone (TSH), proteinuria, and pneumothorax (14, 15). Anlotinib has the potential to keep toxicity, long circulation, and broad-spectrum antitumor activity at manageable levels and is safe and well-tolerated (16).

In our study, the lesion was enlarged after four rounds of preoperative chemotherapy. If complete excision was needed, a wider excision area and a total right lung excision might be required, which in turn might cause major damage to the child as well as impairing her quality of life. Therefore, surgical excision of the tumor was selected, supplemented by 12 rounds of chemotherapy and 28 rounds of radiotherapy in view of the strong possibility of residual tumor. As the lesion was not completely excised, there was a possibility that chemoradiotherapy would not be sufficiently sensitive. Additionally, as the rates of recurrence and metastasis are high in ASPS, there was a possibility for prognosis to be poor. Therefore, the patient's parents were informed of the need for an antiangiogenic drug and its possible side effects. The parents expressed their understanding and agreed to the addition of the antiangiogenic drug anlotinib. The patient whose case is presented in this article is the only case of diagnosed primary pulmonary ASPS in a child to have been treated with an antiangiogenic drug. The combination of surgery with radiotherapy, chemotherapy, and an antiangiogenic agent may reduce the risk of recurrence and metastasis. However, long-term follow-up and observation of prognosis are still needed.

Recently, two cases of patients with ASPS (age at onset: 12 and 17 years) have been reported; these patients have survived 19 years after surgery combined with radiotherapy, chemotherapy, and targeted therapy (17). In our patient, due to a suspected foreign body causing choking, a tracheoscope was used, which enabled early identification of neoplasm without obvious signs of metastasis, following which diagnosis was made. Surgical excision was performed, combined with radiotherapy, chemotherapy, and oral treatment with anlotinib. The prognosis is good, but long-term follow-up is still needed. Taking our case as an example, we speculate that early detection and diagnosis, absence of metastasis at the time of detection, and early complete surgical excision of the tumor may lead to a good prognosis. Follow-up is needed to observe whether active intervention affects the prognosis.

Radical surgical excision of the primary tumor and metastatic lesions is the recommended treatment. Sometimes, radical surgical excision is not feasible in consideration of the child’s quality of life. In cases of pediatric patients with surgically unexcisable lesions, a combination of surgery, chemoradiotherapy, and use of new molecular therapy with antiangiogenic agents as a standard treatment may provide an important reference basis.

## Data Availability

The original contributions presented in the study are included in the article and Supplementary Material; further inquiries can be directed to the corresponding author.

## References

[1] Asiry S Matloob A Khader SN. Alveolar soft part sarcoma: a distinct cytomorphology and characteristic TFE3 staining. Diagn Cytopathol. (2020) 48:684–6. 10.1002/dc.24425 32315502

[2] Tanaka M Homme M Yamazaki Y Shimizu R Takazawa Y Nakamura T. Modeling alveolar soft part sarcoma unveils novel mechanisms of metastasis. Cancer Res. (2017) 77:897–907. 10.1158/0008-5472.CAN-16-2486 27979841

[3] Zhao M Rao Q Wu C Zhao Z He X Ru G. Alveolar soft part sarcoma of lung: report of a unique case with emphasis on diagnostic utility of molecular genetic analysis for TFE3 gene rearrangement and immunohistochemistry for TFE3 antigen expression. Diagn Pathol. (2015) 10:160. 10.1186/s13000-015-0399-5 26369552 PMC4570486

[4] Trabelsi A Ben Abdelkrim S Taher Yacoubi M Mlika S Hmissa S Mokni M Primary alveolar soft part sarcoma of the lung. Rev Mal Respir. (2009) 26:329–32. 10.1016/s0761-8425(09)72591-4 19367208

[5] Kim YD Lee CH Lee MK Jeong YJ Kim JY Park DY Primary alveolar soft part sarcoma of the lung. J Korean Med Sci. (2007) 22:369–72. 10.3346/jkms.2007.22.2.369 17449953 PMC2693611

[6] Brennan B Zanetti I Orbach D Gallego S Francotte N Van Noesel M Alveolar soft part sarcoma in children and adolescents: the European Paediatric Soft Tissue Sarcoma study group prospective trial (EpSSG NRSTS 2005). Pediatr Blood Cancer. (2018) 65:e26942. 10.1002/pbc.26942 29286582

[7] Hagerty BL Aversa J Diggs LP Dominguez DA Ayabe RI Blakely AM Characterization of alveolar soft part sarcoma using a large national database. Surgery. (2020) 168:825–30. 10.1016/j.surg.2020.06.007 32703677 PMC8861880

[8] Falkenstern-Ge RF Kimmich M Wohlleber M Grabner A Friedel G Ott G Lung metastasis of primary alveolar soft-part sarcoma occurring 20 years after initial treatment. Case Rep Oncol Med. (2013) 2013:690520. 10.1155/2013/690520 24379977 PMC3860144

[9] Brahmi M Vanacker H Dufresne A. Novel therapeutic options for alveolar soft part sarcoma: antiangiogenic therapy, immunotherapy and beyond. Curr Opin Oncol. (2020) 32:295–300. 10.1097/CCO.0000000000000652 32541316

[10] Mitton B Federman N. Alveolar soft part sarcomas: molecular pathogenesis and implications for novel targeted therapies. Sarcoma. (2012) 2012:428789. 10.1155/2012/428789 22566752 PMC3337503

[11] Paoluzzi L Maki RG. Diagnosis, prognosis, and treatment of alveolar soft-part sarcoma: a review. JAMA Oncol. (2019) 5:254–60. 10.1001/jamaoncol.2018.4490 30347044

[12] Li S. Anlotinib: a novel targeted drug for bone and soft tissue sarcoma. Front Oncol. (2021) 11:664853. 10.3389/fonc.2021.664853 34094958 PMC8173120

[13] Gao Y Liu P Shi R. Anlotinib as a molecular targeted therapy for tumors. Oncol Lett. (2020) 20:1001–14. 10.3892/ol.2020.11685 32724339 PMC7377159

[14] Syed YY. Anlotinib: first global approval. Drugs. (2018) 78:1057–62. 10.1007/s40265-018-0939-x 29943374

[15] Han B Li K Wang Q Zhang L Shi J Wang Z Effect of anlotinib as a third-line or further treatment on overall survival of patients with advanced non-small cell lung cancer: the ALTER 0303 phase 3 randomized clinical trial. JAMA Oncol. (2018) 4:1569–75. 10.1001/jamaoncol.2018.3039 30098152 PMC6248083

[16] Shen G Zheng F Ren D Du F Dong Q Wang Z Anlotinib: a novel multi-targeting tyrosine kinase inhibitor in clinical development. J Hematol Oncol. (2018) 11:120. 10.1186/s13045-018-0664-7 30231931 PMC6146601

[17] Kuo DJ Menell JS Glade Bender JL. Treatment of metastatic, refractory alveolar soft part sarcoma: case reports and literature review of treatment options in the era of targeted therapy. J Pediatr Hematol Oncol. (2016) 38:e169–72. 10.1097/MPH.0000000000000571 27164526

